# Supplementary material for: Insights on Salt Tolerance of Two Endemic Limonium Species from Spain
Source: Metabolites. 2019 Nov 29;9(12):294. doi: 10.3390/metabo9120294 (PMC6950247; doi:10.3390/metabo9120294)
Supplement: Supplementary file 1 [file metabolites-09-00294-s001.pdf]

Table S1. Metabolic profiles of the two *Limonium* species after 30 days of treatment with the indicated NaCl concentrations. Mean ± SE values are shown (n = 5). Same letters within each column indicate homogeneous groups between treatments for each species according to the Tukey test. ($p < 0.05$).

Sugars	Treatments	Species		
		L. albuferae	*L. dufourii*	*Ratio L. dufourii/L. albuferae*
Erythritol	Control	0.111 ± 0.039 a	0.000 ± 0.000 a	0*
	200 mM NaCl	0.144 ± 0.056 a	0.413 ± 0.074 ab	2.9*
	400 mM NaCl	0.397 ± 0.096 a	0.598 ± 0.133 bc	1.5
	600 mM NaCl	0.312 ± 0.067 a	0.909 ± 0.184 cd	2.9*
	800 mM NaCl	1.000 ± 0.165 b	1.000 ± 0.102 d	1.0
Fructose	Control	0.156 ± 0.026 a	1.000 ± 0.131 b	6.4***
	200 mM NaCl	0.153 ± 0.020 a	0.036 ± 0.007 a	0.2***
	400 mM NaCl	0.436 ± 0.050 ab	0.180 ± 0.051 ab	0.4**
	600 mM NaCl	0.675 ± 0.188 bc	0.320 ± 0.083 ab	0.5
	800 mM NaCl	1.000 ± 0.171 c	0.420 ± 0.064 ab	0.4**
Glucose	Control	0.214 ± 0.066 a	1.000 ± 0.199 b	4.7**
	200 mM NaCl	0.149 ± 0.016 a	0.050 ± 0.011 a	0.3**
	400 mM NaCl	0.402 ± 0.072 a	0.197 ± 0.030 ab	0.5*
	600 mM NaCl	1.000 ± 0.283 b	0.387 ± 0.071 ab	0.4
	800 mM NaCl	0.833 ± 0.170 b	0.884 ± 0.119 ab	1.0
Glycerol	Control	0.472 ± 0.080 a	0.877 ± 0.214 a	1.9
	200 mM NaCl	0.991 ± 0.215 b	0.847 ± 0.158 a	0.9
	400 mM NaCl	0.790 ± 0.157 ab	1.000 ± 0.213 a	1.3
	600 mM NaCl	0.400 ± 0.006 a	0.956 ± 0.194 a	2.4
	800 mM NaCl	1.000 ± 0.188 b	0.892 ± 0.204 a	0.9
Myoinositol	Control	0.625 ± 0.153 ab	1.000 ± 0.195 c	1.6
	200 mM NaCl	1.000 ± 0.251 b	0.893 ± 0.175 bc	0.9
	400 mM NaCl	0.933 ± 0.082 ab	0.711 ± 0.112 abc	0.8
	600 mM NaCl	0.743 ± 0.230 ab	0.526 ± 0.096 ab	0.7
	800 mM NaCl	0.303 ± 0.054 a	0.425 ± 0.063 a	1.4
Raffinose	Control	1.000 ± 0.078 c	0.390 ± 0.172 a	0.4*
	200 mM NaCl	0.797 ± 0.199bc	1.000 ± 0.177 b	1.3
	400 mM NaCl	0.673 ± 0.033 b	0.500 ± 0.106 ab	0.7
	600 mM NaCl	0.152 ± 0.026 a	0.340 ± 0.070 a	2.2
	800 mM NaCl	0.037 ± 0.010 a	0.153 ± 0.059 a	4.1
Rhamnose	Control	0.248 ± 0.030 a	0.327 ± 0.042 a	1.3
	200 mM NaCl	0.281 ± 0.030 a	0.475 ± 0.041 ab	1.7**
	400 mM NaCl	0.495 ± 0.026 b	0.655 ± 0.057 bc	1.3*
	600 mM NaCl	0.693 ± 0.062 c	0.845 ± 0.080 cd	1.2
	800 mM NaCl	1.000 ± 0.102 d	1.000 ± 0.152 d	1.0
Sucrose	Control	0.143 ± 0.011 a	0.202 ± 0.017 a	1.4*

	200 mM NaCl	0.286 ± 0.026 a	0.225 ± 0.046 a	0.8
	400 mM NaCl	0.697 ± 0.062 b	0.359 ± 0.046 a	0.5**
	600 mM NaCl	0.843 ± 0.140 bc	0.686 ± 0.051 b	0.8
	800 mM NaCl	1.000 ± 0.083 c	1.000 ± 0.121 c	1.0

**Organic acids**	**Treatments**	**Species**		
		**L. albuferae**	**L. dufourii**	*Ratio* *L. dufourii/L. albuferae*
Citric acid	Control	0.807 ± 0.186 ab	1.000 ± 0.079 b	1.2
	200 mM NaCl	0.642 ± 0.049 a	0.399 ± 0.013 a	0.6*
	400 mM NaCl	1.000 ± 0.116 b	0.646 ± 0.100 a	0.6
	600 mM NaCl	0.879 ± 0.103 ab	0.646 ± 0.087 a	0.7
	800 mM NaCl	0.888 ± 0.058 ab	0.510 ± 0.010 a	0.6***
Glyceric acid	Control	0.087 ± 0.008 a	0.157 ± 0.028 a	1.8
	200 mM NaCl	0.247 ± 0.063 ab	0.227 ± 0.081 a	0.9
	400 mM NaCl	0.353 ± 0.055 ab	0.277 ± 0.020 a	0.8
	600 mM NaCl	0.327 ± 0.056 b	0.652 ± 0.130 ab	2.0
	800 mM NaCl	1.000 ± 0.130 c	1.000 ± 0.354 b	1.0
Maleic acid	Control	0.943 ± 0.301 a	1.000 ± 0.108 c	1.0
	200 mM NaCl	0.666 ± 0.098 a	0.346 ± 0.097 a	0.5*
	400 mM NaCl	0.843 ± 0.065 a	0.748 ± 0.187 bc	0.9
	600 mM NaCl	0.589 ± 0.183 a	0.465 ± 0.067 ab	0.8
	800 mM NaCl	1.000 ± 0.119 a	0.618 ± 0.154 abc	0.6
Malic acid	Control	0.923 ± 0.414 a	1.000 ± 0.178 b	1.0
	200 mM NaCl	0.402 ± 0.129 a	0.129 ± 0.017 a	0.3
	400 mM NaCl	0.370 ± 0.043 a	0.604 ± 0.184 a	1.6
	600 mM NaCl	0.909 ± 0.122 a	0.242 ± 0.063 a	0.3**
	800 mM NaCl	1.000 ± 0.131 a	0.256 ± 0.054 a	0.3***
Succinic acid	Control	0.407 ± 0.131 a	1.000 ± 0.165 b	2.4*
	200 mM NaCl	0.649 ± 0.119 abc	0.521 ± 0.063 a	0.8
	400 mM NaCl	0.553 ± 0.057 ab	0.775 ± 0.185 ab	1.4
	600 mM NaCl	1.000 ± 0.135 c	0.548 ± 0.080 a	0.6*
	800 mM NaCl	0.841 ± 0.181 bc	0.765 ± 0.188 ab	0.9
Threonic acid	Control	0.102 ± 0.033 a	0.392 ± 0.069 a	3.8*
	200 mM NaCl	0.318 ± 0.044 b	0.524 ± 0.086 ab	1.6
	400 mM NaCl	0.380 ± 0.050 b	0.929 ± 0.184 c	2.4*
	600 mM NaCl	0.760 ± 0.092 c	0.838 ± 0.057 bc	1.1
	800 mM NaCl	1.000 ± 0.075 d	1.000 ± 0.150 c	1.0

Inorganic acid	Treatments	Species		
		**L. albuferae**	**L. dufourii**	***Ratio*** ***L. dufourii/L. albuferae***
Phosphoric acid	Control	0.245 ± 0.128 a	0.505 ± 0.066 ab	2.1
	200 mM NaCl	0.194 ± 0.052 a	0.180 ± 0.041 a	0.9
	400 mM NaCl	0.249 ± 0.039 a	0.551 ± 0.208 ab	2.2
	600 mM NaCl	1.000 ± 0.353 b	0.580 ± 0.186 ab	0.6
	800 mM NaCl	0.750 ± 0.123 b	0.831 ± 0.284 b	1.3

Aminoacids	Treatments	Species		
		L. albuferae	***L. dufourii***	***Ratio*** ***L. dufourii/L. albuferae***
Alanine	Control	0.414 ± 0.034 a	0.619 ± 0.074a	1.5
	200 mM NaCl	0.468 ± 0.089 a	0.681 ± 0.137a	1.5
	400 mM NaCl	1.000 ± 0.132 b	0.806 ± 0.184 a	0.8
	600 mM NaCl	0.616 ± 0.181 a	1.000 ± 0.200 a	1.6
	800 mM NaCl	0.723 ± 0.042 ab	0.879 ± 0.129 a	1.2
Asparagine	Control	0.280 ± 0.129 a	0.176 ± 0.089 a	0.6**
	200 mM NaCl	0.245 ± 0.078 a	0.067 ± 0.007 a	0.3*
	400 mM NaCl	0.137 ± 0.064 a	1.000 ± 0.385 b	7.3
	600 mM NaCl	1.000 ± 0.064 b	0.340 ± 0.171 ab	0.3**
	800 mM NaCl	0.279 ± 0.124 a	0.221 ± 0.048 a	0.8***
Aspartic acid	Control	0.174 ± 0.088 a	0.451 ± 0.180 a	2.6
	200 mM NaCl	0.404 ± 0.153 a	0.549 ± 0.086 a	1.4
	400 mM NaCl	0.349 ± 0.046 a	1.000 ± 0.241 b	2.9*
	600 mM NaCl	1.000 ± 0.155 b	0.476 ± 0.095 a	0.5*
	800 mM NaCl	0.294 ± 0.067 a	0.384 ± 0.055 a	1.3
GABA	Control	0.480 ± 0.044 a	0.586 ± 0.064 ab	1.2
	200 mM NaCl	0.570 ± 0.073 a	0.444 ± 0.129 a	0.8
	400 mM NaCl	0.444 ± 0.098 a	0.909 ± 0.220 ab	2.1
	600 mM NaCl	0.924 ± 0.135 b	0.599 ± 0.057 ab	0.7
	800 mM NaCl	1.000 ± 0.107 b	1.000 ± 0.223 b	1.0
Glutamic acid	Control	1.000 ± 0.186 b	-	-
	200 mM NaCl	0.661 ± 0.168 ab	-	-
	400 mM NaCl	0.610 ± 0.029 a	-	-
	600 mM NaCl	0.949 ± 0.057 ab	-	-
	800 mM NaCl	0.644 ± 0.066 ab	-	-
Glutamine	Control	0.138 ± 0.064 a	-	-
	200 mM NaCl	0.132 ± 0.046 a	-	-
	400 mM NaCl	0.144 ± 0.072 a	-	-

	600 mM NaCl	1.000 ± 0.061 b	-	-
	800 mM NaCl	0.397 ± 0.176 a	-	-
Glycine	Control	0.355 ± 0.044 a	0.523 ± 0.181 ab	1.5
	200 mM NaCl	0.348 ± 0.054 a	0.517 ± 0.083 ab	1.5
	400 mM NaCl	0.476 ± 0.064 ab	1.000 ± 0.216 b	2.1*
	600 mM NaCl	1.000 ± 0.080 c	0.460 ± 0.074 a	0.5**
	800 mM NaCl	0.656 ± 0.098 b	0.383 ± 0.040 a	0.6*
Isoleucine	Control	0.310 ± 0.065 a	0.045 ± 0.021 a	0.2**
	200 mM NaCl	0.409 ± 0.106 ab	0.353 ± 0.085 a	0.9
	400 mM NaCl	0.683 ± 0.114 c	0.823 ± 0.369 b	1.5
	600 mM NaCl	0.632 ± 0.081 bc	0.366 ± 0.032 ab	0.6*
	800 mM NaCl	1.000 ± 0.040 d	0.240 ± 0.021 a	0.2***
Leucine	Control	0.122 ± 0.042 a	0.072 ± 0.035 a	0.6
	200 mM NaCl	0.313 ± 0.111 ab	0.529 ± 0.197ab	1.7
	400 mM NaCl	0.532 ± 0.125 b	0.831 ± 0.367 b	1.9
	600 mM NaCl	0.508 ± 0.048 b	0.377 ± 0.038 ab	0.4
	800 mM NaCl	1.000 ± 0.074 c	0.260 ± 0.025 a	0.3***
Lysine	Control	0.000 ± 0.000 a	0.337 ± 0.054 a	-
	200 mM NaCl	0.258 ± 0.083 ab	0.702 ± 0.182 ab	2.7*
	400 mM NaCl	0.425 ± 0.054 b	0.725 ± 0.216 ab	1.7
	600 mM NaCl	0.955 ± 0.228 c	1.000 ± 0.203 b	1.1*
	800 mM NaCl	1.000 ± 0.142 c	0.792 ± 0.128 ab	0.8
Phenylalanine	Control	0.000 ± 0.000 a	0.081 ± 0.010 a	-
	200 mM NaCl	0.165 ± 0.073 ab	0.128 ± 0.029 a	0.8
	400 mM NaCl	0.211 ± 0.061 b	0.819 ± 0.089 b	4.8**
	600 mM NaCl	0.268 ± 0.044 b	0.225 ± 0.032 a	0.8**
	800 mM NaCl	1.000 ± 0.088 c	0.210 ± 0.030 a	0.2
Proline	Control	0.005 ± 0.001 a	0.004 ± 0.001 a	0.8
	200 mM NaCl	0.113 ± 0.011 a	0.123 ± 0.015 a	1.5
	400 mM NaCl	0.276 ± 0.007 b	0.400 ± 0.047 b	3.6*
	600 mM NaCl	0.680 ± 0.056 c	0.728 ± 0.047 c	1.2*
	800 mM NaCl	1.000 ± 0.062 d	1.000 ± 0.095 d	0.6**
Pyroglutamic	Control	0.217 ± 0.056 a	-	-
	200 mM NaCl	0.231 ± 0.088 a	-	-
	400 mM NaCl	0.122 ± 0.048 a	-	-
	600 mM NaCl	1.000 ± 0.177 b	-	-
	800 mM NaCl	0.448 ± 0.206 a	-	-
Serine	Control	0.210 ± 0.081 a	0.115 ± 0.040 a	0.6
	200 mM NaCl	1.000 ± 0.272 b	0.581 ± 0.079 b	0.6
	400 mM NaCl	0.920 ± 0.089 b	1.000 ± 0.178 c	1.1
	600 mM NaCl	0.970 ± 0.141 b	0.812 ± 0.102 bc	0.8
	800 mM NaCl	0.961 ± 0.055 b	0.577 ± 0.091 b	0.6**

Threonine	Control	0.400 ± 0.068 a	0.132 ± 0.039 a	0.3*
	200 mM NaCl	1.000 ± 0.179 b	0.490 ± 0.079 b	0.5*
	400 mM NaCl	0.903 ± 0.064 b	1.000 ± 0.178 c	1.1
	600 mM NaCl	0.875 ± 0.056 b	0.434 ± 0.059 ab	0.5**
	800 mM NaCl	0.712 ± 0.044 ab	0.257 ± 0.037 ab	0.4***
Tryptophan	Control	0.000 ± 0.000 a	0.020 ± 0.005 a	-
	200 mM NaCl	0.010 ± 0.010 a	0.365 ± 0.059 ab	36
	400 mM NaCl	0.144 ± 0.049 a	1.000 ± 0.245 c	7*
	600 mM NaCl	0.215 ± 0.009 a	0.841 ± 0.156 c	3.2
	800 mM NaCl	1.000 ± 0.428 b	0.399 ± 0.149 bc	0.4
Valine	Control	0.356 ± 0.035 a	0.135 ± 0.024 a	0.4**
	200 mM NaCl	0.453 ± 0.050 ab	0.344 ± 0.093 a	0.8
	400 mM NaCl	0.564 ± 0.033 bc	0.850 ± 0.269 b	1.8
	600 mM NaCl	0.739 ± 0.070 c	0.519 ± 0.043 ab	0.7*
	800 mM NaCl	1.000 ± 0.069 d	0.467 ± 0.041 ab	0.5***

*, **, *** significant at P = 0.05, 0.01 and 0.001 respectively
